# Supplementary figures and images for: Effect of Topical Programmed Death-Ligand1 on Corneal Epithelium in Dry Eye Mouse
Source: Biomolecules. 2024 Jan 4;14(1):68. doi: 10.3390/biom14010068 (PMC10812943; doi:10.3390/biom14010068)

## **Original Westernblot Images**

## Figures

**a. CD4**

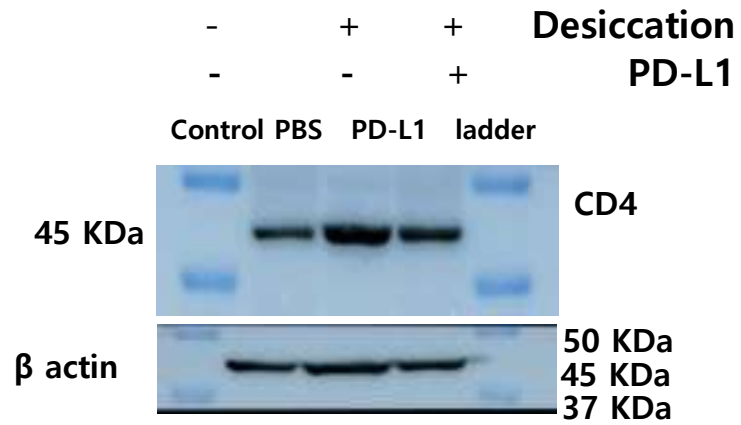

**b. IL-17**

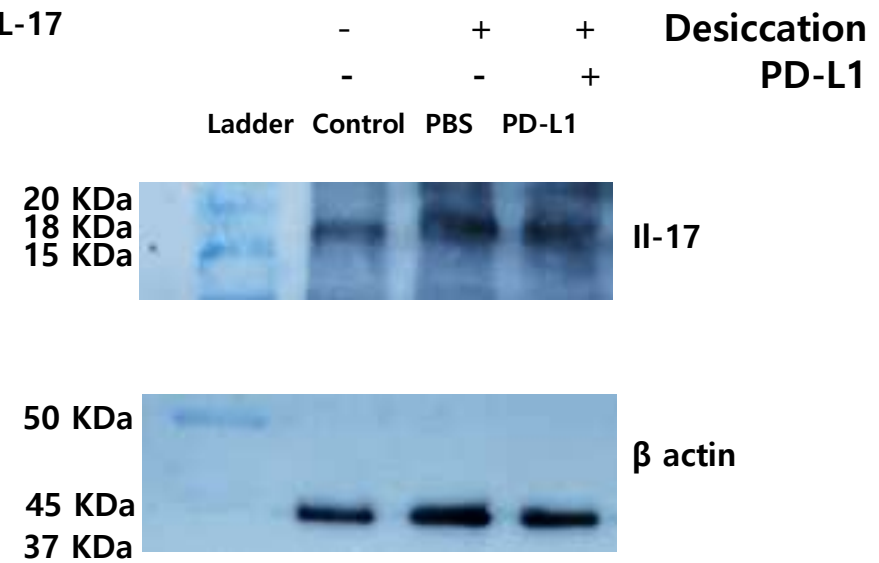

c. pNF-kB

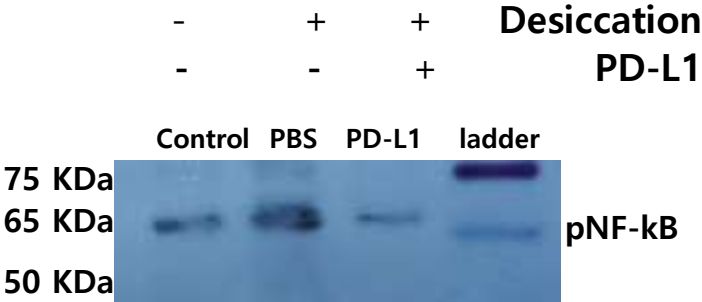

d. p IκB-alpha

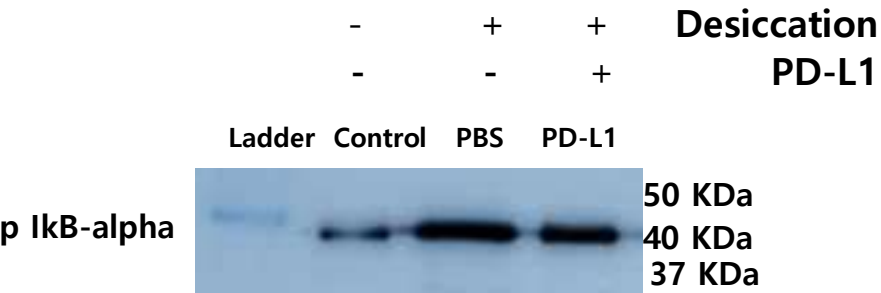

e. NF-kB

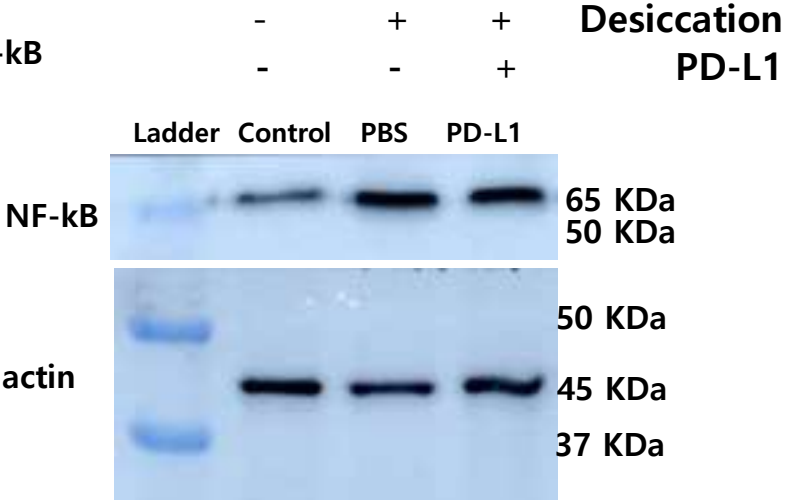

f. IκB-alpha

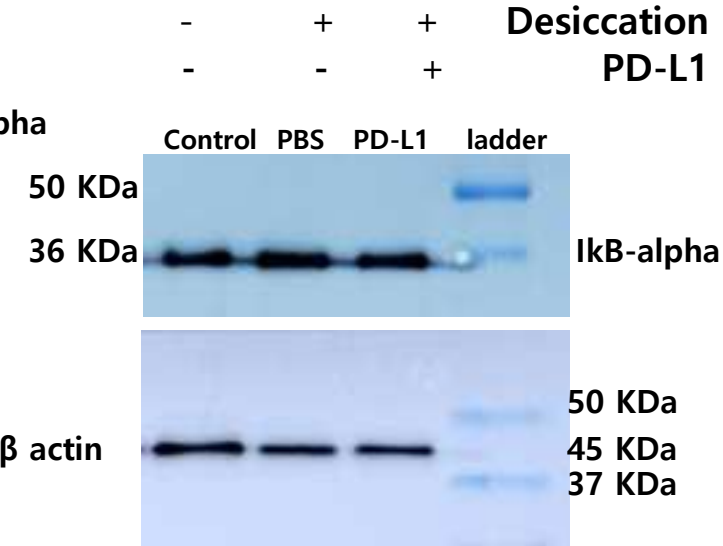

**g. Bax**

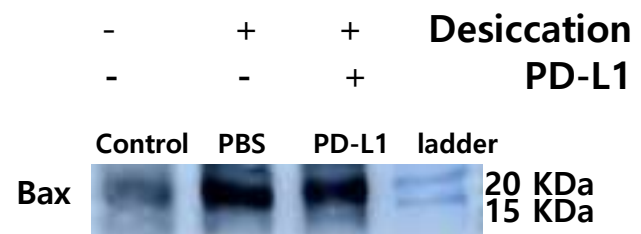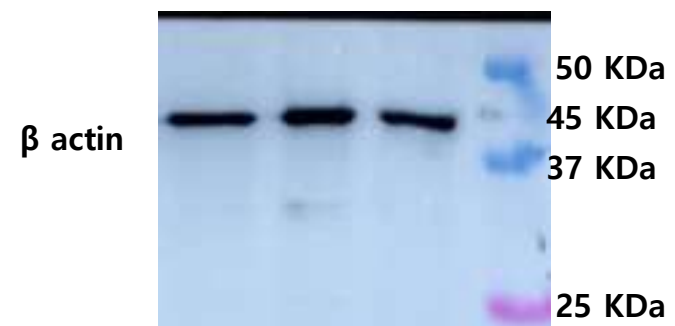

Supplement: Supplementary file 1 [file biomolecules-14-00068-s001.zip › biomolecules-2740661-supplementary.pdf]
